# Use of a Knowledge-Based Governance Approach to Plan a Post-COVID-19 Predoctoral Dental

**DOI:** 10.3390/dj9120142

**Published:** 2021-12-01

**Authors:** Natasha M. Flake, Daniel C. N. Chan, Arthur C. DiMarco, Bruce D. Silverstein

**Affiliations:** 1 Department of Endodontics, University of Washington School of Dentistry, Seattle, WA 98195, USA; 2 Department of Restorative Dentistry, University of Washington School of Dentistry, Seattle, WA 98195, USA; dcnchan@uw.edu (D.C.N.C.); adimarco@ewu.edu (A.C.D.); 3 Department of Dental Hygiene, Eastern Washington University, Spokane, WA 99202, USA; 4 Department of Medicine, University of Washington School of Medicine, Seattle, WA 98195, USA; brucesil@uw.edu; 5 Department of Oral Medicine, University of Washington School of Dentistry, Seattle, WA 98195, USA

**Keywords:** undergraduate dental education, postgraduate dental education, specialty training, teaching methodology, online education, student survey, knowledge-based governance, curriculum

## Abstract

COVID-19 abruptly changed dental education, forcing educators out of their comfort zones and into using new technologies and teaching approaches. At the University of Washington School of Dentistry, a task force evaluated the curricular changes that resulted from COVID and made recommendations for the future predoctoral dental curriculum. This manuscript reports the process employed, the findings of the task force, and how these findings will impact the curriculum. A knowledge-based governance (KBG) approach was employed. KBG focuses on gathering all relevant information and identifying all choices. It separates dialogue from deliberation. Information was gathered via literature review, focus group interviews, electronic surveys, and other metrics. The task force evaluated: (1) delivering didactic content remotely; (2) administering assessments remotely; (3) duplicating preclinical simulation lab courses due to social distancing; and (4) the conversion from a numerical to a credit/no credit grading scale. Key recommendations resulted from focus groups and electronic surveys that allowed any student or faculty member an opportunity to provide input. Some topics were relatively non-controversial and strong recommendations were evident. The most controversial issue was which grading scale should be utilized. A KBG approach is an effective means to address mega issues in the dental school environment.

## 1. Introduction

The COVID-19 pandemic caused dental schools worldwide to change the way they teach students and assess their skills and knowledge. Academicians were forced to significantly rework teaching methodologies and assessment techniques on very short timelines due to dental school closures and social distancing requirements [1,2]. The Association for Dental Education in Europe assessed the initial response of European Academic Dental Institutions to the pandemic and found widespread modifications throughout 69 dental schools [3]. Likewise, dental schools in the United States faced similar challenges and made adjustments to administration, teaching, and learning [4]. Students in one dental school reported they appreciated online learning, but they missed educational experiences and had concerns about independent clinical practice after graduation due to the pandemic [5].

It is likely that some of the curricular changes instigated out of the necessity of the pandemic will find a place in dental school curricula of the future. At the University of Washington School of Dentistry (UWSOD), a task force was charged with evaluating the curricular changes that resulted from the COVID-19 pandemic. The task force evaluated what worked well, what worked poorly, and what aspects should be kept going forward. The following pandemic-induced changes were assessed: (1) delivering didactic courses remotely; (2) administering exams and other assessments remotely; (3) duplicating courses in the preclinical simulation lab due to social distancing requirements; and (4) the conversion to a credit/no credit (Cr/NC) grading scale from a numerical grading scale. This manuscript reports the process employed, the findings of the task force, and how these findings will impact the predoctoral dental curriculum.

## 2. Process

A knowledge-based governance (KBG) approach was employed to assess the curricular changes triggered by COVID-19 [6,7,8]. KBG is a philosophy of governance and decision-making used by professional associations. It values the quality of the information on which decisions are made, rather than who makes the decisions. It focuses on gathering all relevant information and identifying all choices, rather than focusing on the conclusion. The advantages and disadvantages of each choice are considered. KBG separates dialogue from deliberation. Dialogue seeks to inform and ensure common understanding of the information that impacts a decision, including consideration of all available options. Deliberation focuses on evaluation, decisions, and conclusions.

A KBG process asks four key questions [6,8]. Adapted for the dental school setting, these are: (1) What do we know about our stakeholders’ needs, wants, and preferences that is relevant to this decision? (2) What do we know about the current reality and evolving dynamics of our school and profession that is relevant to this decision? (3) What do we know about the capacity and strategic position of our school that is relevant to this decision? (4) What are the ethical implications of our choices?

The task force prioritized the success of the school as a whole over individual agendas and was motived by the long-term future, not just the next academic year. To achieve this, a four-pronged approach to information gathering was implemented: (1) literature review; (2) focus group interviews; (3) surveys; and (4) metrics.

Thirteen focus group and individual interviews were conducted (Table 1). Participation by all stakeholders was voluntary. Student recruitment for focus group participation was facilitated by student leaders. Focus groups were held online using the Zoom platform. Standardized questions addressing all topics of interest were used for the interviews of students, foundations faculty, preclinical faculty, Clerkship Directors, and Associate Deans. Questions specific to staff expertise were used for staff interviews. Staff were interviewed who had knowledge and experience in scheduling, information technology, educational technology, and managing preclinical laboratory courses. Questions for the Graduate Program Directors focused on grading scales and graduate program admissions.

Electronic surveys were sent to all students, faculty, and Graduate Program Directors. Survey questions were based on comments heard in the focus groups. Standardized questions addressing all topics were used for student and faculty surveys, with a few questions that differed or differed in wording depending on the target audience. The Graduate Program Directors survey specifically addressed the impact of grading scales on graduate program admissions. All surveys consisted of a series of statements grouped by topic, and participants were instructed to rate their level of agreement with the statement as: “strongly agree”, “agree”, “neutral”, “disagree”, “strongly disagree”, or “not applicable/don’t know”. All electronic surveys also invited open-ended comments. Course directors were also asked for any metrics that could compare their pre-COVID-19 and COVID-era course to provide supporting data that might aid in formulating recommendations.

## 3. Findings

We report the results of the surveys and relate them to the focus group findings and metrics from course directors. Survey response demographics are reported in Table 2. For the narrative, we considered responses of “strongly agree” and “agree” as supporting a statement, and responses of “neutral”, “disagree”, “strongly disagree”, and “not applicable/don’t know” as not supporting a statement. The complete results of the survey are detailed in Appendix A. The recommendations of the task force are included in the Discussion section.

### 3.1. Delivering Didactic Courses Remotely

The UWSOD academic calendar is based on academic quarters. All in-person courses ceased in the last week of winter quarter 2020 due to the pandemic. Didactic courses have remained remote since that time, with an anticipated return to in-person courses in autumn quarter 2021.

**Figure 1:**
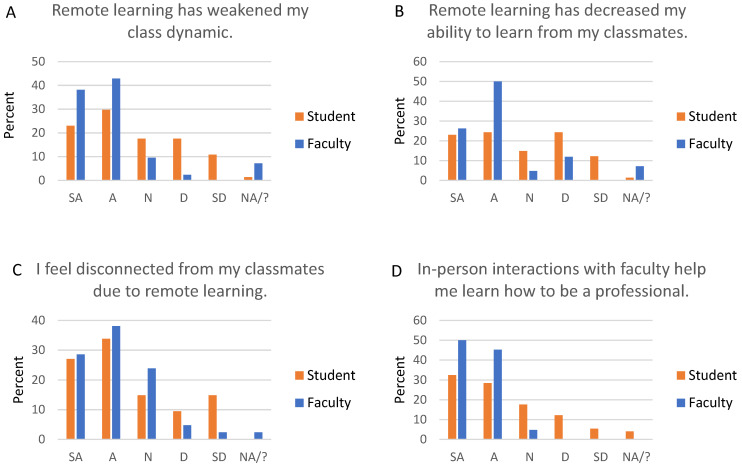
Social impacts of remote learning. All students and faculty were asked about the social impacts of remote learning. Graph titles reflect wording asked of students, but wording was customized for students and faculty. For example, the statement for students was “Remote learning has weakened my class dynamic”, and the statement for faculty was “Remote learning has weakened dental student class dynamics”. SA, strongly agree; A, agree; N, neutral; D, disagree; SD, strongly disagree; NA/?, not applicable/don’t know.

Students and faculty share concerns about the social impacts of remote learning (Figure 1): 81% of faculty and 53% of students agree or strongly agree that remote learning has impacted class dynamics (Figure 1A). Sixty-one percent of students feel disconnected from their classmates, and 45% feel disconnected from faculty, while 67% of faculty feel disconnected from students (Figure 1C). The social effects also impact learning: 76% of faculty and 47% of students think that remote learning decreases student ability to learn from classmates (Figure 1B). Faculty (95%) and students (61%) agree that in-person interactions with faculty promote professional development (Figure 1D).

The majority of faculty generally prefer in-person didactics. Faculty understand student level of comprehension and can “read the room” and adjust teaching in real-time better when didactics are in person. They find that students are more likely to ask a question or speak up in person, and that students do not actively engage in Zoom sessions. While only 7% of faculty generally prefer pre-recorded asynchronous lectures, 55% like the flexibility of this format. They do think it takes more time to prepare a quality pre-recorded lecture than to deliver an in-person lecture. Faculty also are concerned about students not viewing assigned lectures, which can be corroborated by statistics in the learning management software.

Students are more split than faculty, with 39% generally preferring in-person didactics, and 51% preferring pre-recorded asynchronous lectures. Approximately half (51%) of students find it difficult to focus during Zoom sessions, and 27% say they log on but do not typically actively engage. One-third (34%) of students are more likely to ask a question or speak up during an in-person didactic session. A large majority (88%) of students like the flexibility of asynchronous pre-recorded lectures, though over half (55%) also like to be able to ask questions in real-time.

The class of 2021 focus group, the most experienced student cohort, unanimously agreed that pre-recorded asynchronous lectures are convenient, but that in-person formats are best for learning. They stated that pre-recorded lectures are fine if the goal is to pass a test, but in-person interactions are best for learning to become a dentist. These students explained that in-person didactics are more organic and foster more questions, discussion, and learning.

### 3.2. Administering Assessments Remotely

Almost all faculty adapted their exam format for remote administration, but the adaptations varied. For example, some faculty increased the number of essays, while others increased the number of multiple-choice questions. Some faculty proctored exams remotely via Zoom or via automated software; others did not proctor and relied on the honor system or gave open-book exams.

Approximately one-half (52%) of faculty think students study less when an exam is open-book, and one-third (33%) think students learn less when an exam is open-book. From the student perspective, 38% say they study less, and 22% say they learn less when an exam is open-book.

Importantly, 62% of faculty and 32% of students are concerned about academic dishonesty when exams are not proctored. Students are more comfortable than faculty with remote proctoring, but still only 41% of students are comfortable with a Zoom proctor and 28% with proctoring by automated software. Twenty-seven percent of students say it is difficult to find a suitable (quiet, private) space to take a remote exam.

### 3.3. Split Shift in the Preclinical Simulation Lab

Social distancing requirements allow only one-half of a class in the preclinical simulation lab at once. Thus, all courses in the preclinical lab are held in two shifts. In addition, lectures, quizzes and exams are prohibited in the preclinical lab and must be completed remotely. This necessitates a complex scheduling puzzle, where one-half of the class attends lab and the other half of the class remains offsite during any class period.

Feedback from course directors strongly supports a return to the entire class in the lab at once. Course directors adapted to the split shift in varied ways, including covering some content only didactically, shifting content to other courses, focusing on hands-on experience at the expense of didactics, and increasing student practice time after hours. The most compelling reason to return to a single lab session is the reduction in time for hands-on experience during class for students with the split shift. Further, scheduling for the split shift is a logistical challenge that impacts other courses. From the student perspective, 65% feel disconnected from classmates who do not share the same lab time, and 31% are concerned about imbalanced experiences between the two groups of students.

### 3.4. Conversion to Credit/No Credit Grading from a Numerical (4.0) Grading Scale

The UWSOD historically utilizes a numerical (4.0) grading scale for most required predoctoral dental courses and calculates a class rank based on academic performance. Starting spring quarter 2020, predoctoral courses were temporarily converted to a Cr/NC (i.e., pass/fail) grading scale. Courses are scheduled to return to their pre-COVID-19 grading scales in autumn quarter 2021. The rationale for Cr/NC grading was primarily due to concerns with remote learning. Concerns for students included internet connectivity difficulties, family responsibilities, and discrepancies among students in their housing and economic resources. Apprehension over the ability to verify academic integrity with distance learning also contributed. Overall, Cr/NC grading was intended to alleviate stress and help students cope with the uncertainties of the time. Given the circumstances, the decision to temporarily implement Cr/NC grading had wide, though not universal, support from both students and faculty.

**Figure 2:**
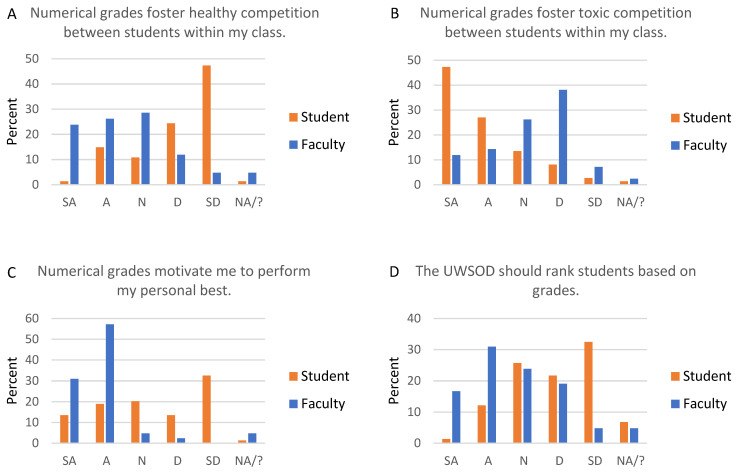

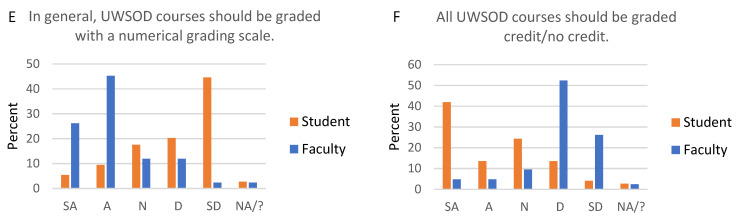
Faculty and student responses regarding grading scales. All students and faculty were asked about numerical and credit/no credit grading scales. Graph titles reflect wording asked of students, but wording was customized for students and faculty. For example, the statement for students was “Numerical grades motivate me to perform my personal best”, and the statement for faculty was “Numerical grades motivate students to perform their personal best”. SA, strongly agree; A, agree; N, neutral; D, disagree; SD, strongly disagree; NA/?, not applicable/don’t know; UWSOD, University of Washington School of Dentistry.

The issue of Cr/NC versus 4.0 grading scales was the most polarizing subject the task force addressed. Individual stakeholders have strong and diverse opinions on the subject. From the perspective of class culture, 50% of faculty think numerical grades foster healthy competition, and 26% think numerical grades foster toxic competition within a class (Figure 2A,B). In contrast, 16% of students think numerical grades foster healthy competition, and 74% think numerical grades foster toxic competition (Figure 2A,B). Further, 74% of students are more likely to collaborate with classmates when a course is graded Cr/NC.

From an academic perspective, faculty strongly prefer numerical grades. The differential effort that students put into courses with a numerical versus Cr/NC grading scale was raised by both faculty and students. Historically, when students had Cr/NC and 4.0 scale courses concurrently, they put less effort into Cr/NC courses, and even stopped attending once they had earned enough points to pass a Cr/NC course. Most faculty (88%) believe numerical grades motivate students to perform their personal best (Figure 2C). Ninety percent say students study more, and 57% say students learn more in a numerically-graded course. Overall, 71% of faculty think that in general, courses should be graded with a numerical grading scale, while only 10% think all courses should be Cr/NC (Figure 2E,F). Finally, 76% of faculty agree or strongly agree that numerical grades foster academic excellence.

From an academic perspective, students favor Cr/NC grades, though not quite as strongly as faculty favor numerical grades. Approximately one-third (32%) of students say numerical grades motivate them to perform their personal best (Figure 2C). Approximately one-third (32%) of students study more, and 14% say they learn more, in a course with a numerical grade. Overall, 15% of students think that in general, courses should be graded with a numerical grading scale, while 55% feel all courses should be graded Cr/NC (Figure 2E,F).

A common concern about Cr/NC grading is its impact on graduate program admissions. Graduate Program Directors reported they consider both GPA and class rank in the admissions process. They relate it is difficult to assess applicants who went to a dental school that does not issue grades. All Graduate Program Directors who responded are concerned that a switch to Cr/NC grading might impact student matriculation to graduate programs.

Class rank can be considered separately from grades, as it is possible to calculate grade point average but not student rank. Forty-eight percent of faculty think students should receive a class rank based on grades (Figure 2D). One-fifth (20%) of students are motivated by class rank, and 14% feel that the school should rank students (Figure 2D). However, 5 of 8 Graduate Program Directors say if they could have only one piece of objective data for applicants, it would be class rank.

Each class of students has a different perspective, since they have experienced different time and grading formats. Even the class of 2021, who have the most experience and yet no personal stake in the decision, were conflicted. Students in this focus group were split on whether they preferred Cr/NC versus numerical grades; however, they agreed they studied less, and probably learned less, for a Cr/NC course.

## 4. Recommendations and Discussion

### 4.1. Delivering Didactic Courses Remotely

The task force recommends that the method of delivering didactic content ultimately be decided by course directors. In-person interactions and learning are highly valued. Faculty and students agree that personal interactions with faculty help students learn to be professionals, the “hidden” curriculum [9]. However, some content can be effectively delivered remotely, and some courses or content within a course are more amenable to remote delivery. The strengths and limitations of remote learning cited by students are similar to those reported by students in other countries [10,11,12].

Asynchronous remote didactics have the advantage of offering students multiple, flexible review opportunities. In cases where content is delivered by pre-recorded lectures or other asynchronous methods, the task force recommends they be followed-up with interactive sessions such as question and answer sessions, case reviews, etc. The task forces also recommends checkpoints throughout a course to ensure students are learning content in a timely manner (e.g., quizzes or other exercises).

A clearly successful example of the use of synchronous remote learning at UWSOD is the Regional Initiatives in Dental Education (RIDE) program. Synchronous remote methods have been used successfully with the RIDE program for many years, where dental students in Spokane, Washington actively participate in courses that are delivered in Seattle. Students are together in a classroom with an instructor in both Seattle and Spokane. This differs from the remote synchronous didactics started during the pandemic, which are often experienced as lecturing to “black boxes” on Zoom. With some exceptions, the task force has little enthusiasm for synchronous remote lectures, due to low student engagement. These methods also lack the flexible timing offered by asynchronous methods.

For all methods, the task force recommends that faculty be trained in effective teaching, and that they have protected time to dedicate to this training. Though nearly all the feedback received on remote asynchronous didactics related to pre-recorded lectures, other modalities are available. There is ample evidence that online teaching can be effective in higher education [13]. It is a matter of identifying which methods are useful for individual courses and providing faculty the training and time to implement these methods.

### 4.2. Administering Assessments Remotely

The task force strongly recommends that exams be administered in person under proctored conditions. The convenience of administering exams remotely does not outweigh the concerns over academic integrity, either real or perceived. Exceptions to this recommendation would be those exam formats that were administered as take-home exams prior to COVID.

### 4.3. Split Shift in the Preclinical Simulation Lab

The task force strongly recommends that the preclinical lab courses return to all students attending at the same time. The only recognized advantages of the split shift are potential lower student:faculty and/or student:equipment ratios. Improved ratios could also be achieved by deploying additional faculty or procuring additional equipment for preclinical courses, though this may be unfeasible.

### 4.4. Conversion to Credit/No Credit Grading from a Numerical (4.0) Grading Scale

The advantages and disadvantages of both Cr/NC and numerical grading scales are compelling. The task force recommends that the SOD resume its pre-COVID-19 grading scales for the foreseeable future. This means a numerical grading scale for most courses, with exceptions for courses that were graded Cr/NC prior to the pandemic. The task force recommends that, if a global change to Cr/NC grading is to be considered, more deliberate preparation is needed. Namely, a change to Cr/NC would require: (1) a culture that emphasizes excellence, rather than passing, and a mindset that the objective of the curriculum is to prepare students to be clinicians rather than test takers; and (2) faculty buy-in.

The attitudes of faculty and students regarding Cr/NC grading reflect those described in the literature [14]. In a point/counterpoint article, Jham, Cannella, and Abdibi support the position that a pass/fail system improves learning experiences for students [15]. They argue that pass/fail grades positively impact students’ psychological well-being, that academic performance of health care students can be successfully evaluated using pass/fail grades, and that pass/fail grades promote self-directed and collaborative learning [15]. Austin, Allareddy, and Petrie support the position that a traditional grading system provides more objectivity and reliability in student evaluation [15]. They argue that traditional grades motivate student performance, provide objectivity and validity in performance assessment, and are important objective criteria for evaluation of graduate program applicants [15]. Ramaswamy et al. argue in favor of pass/fail grading, citing promotion of student well-being, intrinsic motivation, and competency-based education [16]. Further, comments provided by students in our focus groups and surveys are consistent with those of other dental students [17,18,19].

The question of how advanced dental education programs can best evaluate candidates has increased in importance in the past decade with the change to pass/fail scores on national board exams, particularly for applicants who attend a pass/fail dental school [20,21]. The Advanced Dental Admission Test (ADAT) was developed to provide a metric for use in graduate program admissions [22]. Each graduate program has its own process, but program directors consider many interacting factors when selecting candidates for graduate programs. The Graduate Program Directors reported they consider both GPA and class rank in the admissions process, consistent with published literature [23,24,25]. Clinical grades, dental school class rank, and dental school GPA were the three most important factors considered by pediatric dentistry program directors [24]. Among endodontic program directors, dental school class rank was the second most important factor, while dental school clinical grades, endodontic grades, and basic science grades were ranked sixth through eighth in importance [23]. For periodontics graduate programs, dental school clinical grades and dental school periodontics grades ranked second and third, while dental school class rank was the seventh most important factor considered in admissions [25].

### 4.5. Limitations

The KBG approach was effective overall, though there are limitations associated with the methods employed. First, a limitation of the electronic surveys is the risk of response bias, particularly because participants likely understood the purpose of the survey. Second, course directors were asked for any metrics that could be used to compare their course in pre-COVID-19 and COVID-19 times. We were aware that there would be caveats associated with any data collected. With the method of delivering content, the assessment method, and the grading scale changing simultaneously in most cases, it would be difficult to tease out the individual impact of these factors. Third, different cohorts of students have different lived experiences in dental school. For example, students in the class of 2024 provided input on a grading scale that they had not experienced in dental school, as pandemic-related changes occurred prior to their matriculation. In comparison, the class of 2021 had the greatest experience with different methods of teaching and learning in dental school and had the least personal stake in any decision about the curriculum. Although the focus group for this class was extremely insightful, the class also had the lowest participation rate in the electronic survey. A final caveat of note is the timeline of the process related to the course of the pandemic. During the time of information gathering and analysis, pandemic-related restrictions at the University were relaxed, followed by a return to more restrictive social distancing rules. This highlights the need for dental schools to have alternative educational methods in reserve and ready to implement as needed due to fluctuations in the pandemic or other impactful factors.

## 5. Conclusions

Use of the KBG approach to plan changes to a predoctoral dental curriculum was successful. Focus groups followed-up by electronic surveys allowed any student or faculty member the opportunity to provide input. Focus group and individual interviews also facilitated input from staff with valued expertise relevant to the decisions at hand. Some topics were relatively non-controversial and strong recommendations were evident, while other topics generated more divisive opinions. The KBG approach allowed for transparency and assurance of thorough vetting of difficult decisions. A KBG approach is an effective means to address mega issues in the dental school environment.

## Figures and Tables

**Table 1:** Focus group interviews. Standardized questions were asked for the student, foundations faculty, preclinical faculty, Clerkship Directors, and Associate Dean interviews. Questions specific to expertise were asked for staff and Graduate Program Directors interviews.

Students	Faculty
Class of 2021	Foundations faculty
Class of 2022	Preclinical faculty
Class of 2023	Clerkship Directors
Class of 2024	Graduate Program Directors
Staff	Administration
Scheduler	Associate Dean of Academic Affairs and
Director of Information Technology Educational Technology Specialist Preclinical Lab Support	Associate Dean of Student Services and Admissions

**Table 2:** Demographics of electronic survey responses. Age and gender identity were asked as optional, open-ended questions. Regular appointment faculty include both full-time and part-time faculty. Affiliate faculty volunteers are paid a nominal stipend, typically one or less day per week.

Faculty Demographics	Student Demographics
Total faculty responses, n = 42	Total student responses, n = 74
Regular faculty appointments, n = 33	Class of 2021, n = 8
Affiliate faculty appointments, n = 9	Class of 2022, n = 23
Time worked in dental education:	Class of 2023, n = 12
0–5 years, n = 8	Class of 2024, n = 31
6–10 years, n = 4	Students reporting age, n = 55
11–20 years, n = 10	Median age = 25 years
>20 years, n = 20	Mean age = 26 years
Teach in these predoc settings:	Age range = 21–44 years
Foundations, n = 9	Students reporting gender identity, n = 55
Preclinical dental courses, n = 21	Female/woman, n = 32
Clinical dental courses, n = 21	Male/man, n = 22
Clinic, n = 18	Genderqueer/non-binary, n = 1
Other, n = 1	
Do not teach predoc, n = 3	
Teach in >1 predoc setting, n = 18	
Course director for predoc course, n = 22	
Faculty reporting age, n = 30	
Median age = 57 years	
Mean age = 55 years	
Age range = 29–75 years	
Faculty reporting gender identity, n = 29	
Female/woman, n = 13	
Male/man, n = 16	

## Data Availability

Not applicable.

## Appendix A

Electronic survey results. Standardized questions were asked for the all-student and all-faculty surveys. Questions specific to graduate program admissions were asked on the Graduate Program Directors surveys. Data represent the number of respondents who answered with each response.

**Table A1:** Electronic survey results.

ALL-FACULTY AND ALL-STUDENT SURVEY RESULTS						
**Statements Related to Social Impacts of Remote Learning.**						
	**SA**	**A**	**N**	**D**	**SD**	**NA/?**
**FACULTY**						
Remote learning has weakened dental student class dynamics.	16	18	4	1	0	3
Remote learning has decreased student’s ability to learn from their classmates.	11	21	2	5	0	3
I feel disconnected from students due to remote learning.	12	16	10	2	1	1
In-person interactions with faculty help students learn how to be a professional.	21	19	2	0	0	0
**STUDENT**						
Remote learning has weakened by class dynamic.	17	22	13	13	8	1
Remote learning has decreased my ability to learn from my classmates.	17	18	11	18	9	1
I feel disconnected from my classmates due to remote learning.	20	25	11	7	11	0
I feel disconnected from faculty due to remote learning.	20	13	16	17	8	0
I have formed study groups during remote learning.	13	19	10	18	12	2
In-person interactions with faculty help me learn how to be a professional.	24	21	13	9	4	3
**Statements related to didactics. Didactics can be lectures or small group seminars/discussions. Didactics can be in-person, remote synchronous (i.e., Zoom), or remote asynchronous (i.e., pre-recorded).**						
	**SA**	**A**	**N**	**D**	**SD**	**NA/?**
**FACULTY**						
Students log on to my Zoom sessions, but typically do not actively engage in them.	5	11	8	5	2	11
I prefer students to have their cameras ON during Zoom sessions.	18	11	4	0	1	8
Technical difficulties often hinder my Zoom sessions.	1	4	5	18	5	9
I understand students’ level of comprehension better when didactics are in-person.	13	17	3	2	3	4
I am able to read the room and adjust my teaching in real time better during in-person didactics.	16	19	1	1	0	5
Students are more likely to ask questions or speak up during an in-person didactic session (versus a Zoom session).	14	15	3	2	1	6
I like students to be able to ask questions in real time.	19	18	3	0	0	2
I appreciate the flexibility of pre-recorded asynchronous lectures.	6	17	5	5	2	7
It takes me longer to prepare a quality pre-recorded lecture than it does to deliver an in-person lecture.	15	7	7	6	0	7
In general, I prefer in-person didactics.	17	13	6	3	1	2
In general, I prefer pre-recorded asynchronous lectures (versus in-person lectures or Zoom lectures).	0	3	11	15	10	3
**STUDENT**						
I log on to Zoom sessions, but typically do not actively engage in them.	22	16	12	15	9	0
It is difficult to focus during Zoom sessions.	4	16	12	30	12	0
I prefer to have my camera OFF during Zoom sessions.	19	29	20	6	0	0
I often have technical difficulties that hinder Zoom sessions.	1	5	10	27	31	0
Faculty understand my level of comprehension better when didactics are in-person.	16	20	11	10	9	8
I am more likely to ask a question or speak up during an in-person didactic session (versus a Zoom session).	16	9	7	18	21	3
I like the ability to ask questions in real time.	19	22	22	9	2	0
I appreciate the flexibility of pre-recorded asynchronous lectures.	47	18	6	2	1	0
In general, I prefer in-person didactics.	19	10	14	12	17	2
In general, I prefer pre-recorded asynchronous lectures (versus in-person lectures or Zoom lectures).	26	12	19	10	7	0
**Statements related to remote exams. This could also mean quizzes.**						
	**SA**	**A**	**N**	**D**	**SD**	**NA/?**
**FACULTY**						
Students study less when an exam is open-book.	10	12	8	6	1	5
Students learn less when an exam is open-book.	4	10	14	7	2	5
I have concerns about academic dishonesty when exams are not proctored.	8	18	5	6	0	5
I am comfortable with the use of remote proctoring via a Zoom proctor.	1	6	9	6	6	14
I am comfortable with the use of remote proctoring via automated software (e.g., Proctorio).	1	5	9	7	5	15
**STUDENT**						
I study less when an exam is open-book.	6	22	10	28	8	0
I learn less when an exam is open-book.	6	10	10	29	19	0
I have concerns about academic dishonesty when exams are not proctored.	8	16	17	20	12	1
I am comfortable with remote proctoring via a Zoom proctor.	7	23	15	18	11	0
I am comfortable with remote proctoring via automated software (e.g., Proctorio).	4	17	15	14	22	2
It is difficult for me to find a suitable (quiet, private) space to take a remote exam.	11	9	11	25	18	0
**Statements related to course grading schemes: numerical grades (4.0 scale) versus credit/no credit grades.**						
	**SA**	**A**	**N**	**D**	**SD**	**NA/?**
**FACULTY**						
Numerical grades foster healthy competition between students within a class.	10	11	12	5	2	2
Numerical grades foster toxic competition between students within a class.	5	6	11	16	3	1
Numerical grades motivate students to perform their personal best.	13	24	2	1	0	2
Credit/no credit grades motivate students to perform “good enough”.	8	18	11	2	2	1
Students study more in a course with a numerical grading scale.	16	22	2	0	0	2
Students learn more in a course with a numerical grading scale.	6	18	10	1	3	4
I am concerned credit/no credit grades may impact students’ post-graduation plans.	11	16	7	3	2	3
Students are more likely to contest individual points on an assessment in a course with a numerical grading scale.	12	22	3	1	0	4
I am concerned about calibration of graders in a course with a numerical grading scale.	5	21	9	4	0	3
Numerical grades foster academic excellence at the UWSOD.	11	21	5	3	0	2
The UWSOD should rank students based on grades.	7	13	10	8	2	2
In general, UWSOD courses should be graded with a numerical grading scale.	11	19	5	5	1	1
All UWSOD courses should be graded credit/no credit.	2	2	4	22	11	1
**STUDENT**						
Numerical grades foster healthy competition between students within my class.	1	11	8	18	35	1
Numerical grades foster toxic competition between students within my class.	35	20	10	6	2	1
Numerical grades motivate me to perform my personal best.	10	14	15	10	24	1
Credit/no credit grades motivate me to perform “good enough”.	6	18	7	17	24	2
I study more in a course with a numerical grading scale.	7	17	12	13	21	4
I learn more in a course with a numerical grading scale.	6	4	10	22	29	3
I am concerned credit/no credit grades may impact my post-graduation plans.	4	9	13	16	30	2
I am more likely to contest individual points on an assessment in a course with a numerical grading scale.	36	18	10	6	4	0
I am concerned about calibration of graders in a course with a numerical grading scale.	36	22	9	2	1	4
The UWSOD should rank students based on grades.	1	9	19	16	24	5
I am motivated by class rank.	4	11	10	12	37	0
I am more likely to collaborate with my classmates when a course is graded credit/no credit.	30	15	12	12	3	2
I typically study for the grade in a course with a numerical grading scale, rather than studying to become a good dentist.	26	24	5	11	7	1
In general, UWSOD courses should be graded with a numerical grading scale.	4	7	13	15	33	2
All UWSOD courses should be graded credit/no credit.	31	10	18	10	3	2
**Statements related to courses in the preclinical simulation lab.**						
	**SA**	**A**	**N**	**D**	**SD**	**NA/?**
**FACULTY**						
There is a disconnect between students in the two lab groups.	3	4	2	2	1	30
I am concerned about imbalanced experiences between two groups of students in the lab.	1	3	4	4	1	29
Students get greater personal attention from faculty with the split shift in the lab.	3	7	3	0	2	27
It is easier for students to use shared equipment with the split shift in the lab.	2	7	3	0	1	29
I spend more time preparing and teaching with the split shift in the lab.	3	3	3	2	0	31
Teaching the same course twice detracts from my other responsibilities as a faculty member.	5	9	3	3	0	22
**STUDENT**						
I feel disconnected from classmates who do not share the same lab time as me.	32	16	5	6	3	12
I am concerned about imbalanced experiences between two groups of students in the lab.	9	14	11	17	9	14
I get greater personal attention from faculty with the split shift in the lab.	20	16	6	3	4	25
It is easier to use shared equipment with the split shift in the lab.	26	17	5	2	2	22
**GRADUATE PROGRAM DIRECTOR SURVEY RESULTS**						
	**SA**	**A**	**N**	**D**	**SD**	**NA/?**
I consider GPA when assessing applicants to my graduate program.	4	4	0	0	0	0
I consider class rank when assessing applicants to my graduate program.	5	2	1	0	0	0
It is difficult to assess an applicant who went to a dental school that does not issue grades.	4	4	0	0	0	0
I prefer that applicants to my graduate program report GPA.	6	2	0	0	0	0
I prefer that applicants to my graduate program report class rank.	6	2	0	0	0	0
Class rank is a poor metric for applicants, because students within a class are separated by extremely small differences in GPA.	0	1	3	3	1	0
Class rank is a valuable metric for applicants, because GPAs are elevated by grade inflation.	2	3	1	1	0	1
I am concerned that a switch to credit/no credit grades at UWSOD may impact our students’ matriculation to graduate programs.	3	5	0	0	0	0
I am concerned that eliminating class rank at UWSOD may impact our students’ matriculation to graduate programs.	4	3	0	1	0	0
	**Class rank**		**GPA**		**Standardized test score**	
If you could have only one piece of objective data when assessing applicants to your graduate program, which would it be?	5		3		0	
